# Urachal adenocarcinoma with cervical invasion misdiagnosed as primary cervical adenocarcinoma: a case report and literature review

**DOI:** 10.3389/fonc.2024.1410291

**Published:** 2024-09-27

**Authors:** Yiran Wang, Maomao Li, Kaixuan Yang, Qingli Li, Ping Wang

**Affiliations:** ^1^ Department of Gynecology and Obstetrics, West China Second University Hospital, Sichuan University, Chengdu, Sichuan, China; ^2^ Key Laboratory of Birth Defects and Related Diseases of Women and Children (Sichuan University), Ministry of Education, Chengdu, Sichuan, China; ^3^ Department of Pathology, West China Second University Hospital, Sichuan University, Chengdu, Sichuan, China

**Keywords:** urachal carcinoma, cervical cancer, histopathological examination, anterior pelvic exenteration, case report

## Abstract

**Background:**

Urachal carcinoma (UrC) is a rare malignancy with no known specific early symptoms. It is often diagnosed at advanced stages and is associated with poor prognosis.

**Case presentation:**

This study presents a rare case of urachal adenocarcinoma (UrAC) invading the bladder and vagina in a female patient. Initially, the patient was misdiagnosed as having a primary cervical adenocarcinoma 2.5 years prior. Subsequently, anterior pelvic exenteration and bilateral ureterocutaneostomies were performed. Twenty months after the first surgery, the patient was diagnosed with rectal metastasis and received gemcitabine chemotherapy. After achieving a stable disease state, the patient underwent laparoscopic ultralow rectal anterior resection, ultralow anastomosis of the sigmoid colon and rectum, prophylactic transverse colostomy, and right common iliac and external iliac lymph node dissection. The patient then received a cycle of postoperative chemotherapy with oxaliplatin and capecitabine; however, treatment was stopped due to adverse reactions. The patient continues to receive regular follow-ups, and her general condition is good.

**Conclusions:**

UrC is rare, and preoperative differential diagnosis is difficult. This is the first report of UrC being misdiagnosed as cervical cancer. The presented case highlights the importance of accurate histopathological examination and comprehensive analysis. Anterior pelvic exenteration was also identified as a potentially effective treatment strategy for patients with local pelvic recurrence of UrC, although further investigation is required.

## 1 Introduction

Urachal carcinoma (UrC) is a rare non-urothelial malignancy that accounts for less than 1% of all bladder cancers (1). Adenocarcinoma is the most common type of UrC (2), which generally occurs along the midline from the apex vesicae to the umbilicus and within the Retzius space (3). The most common symptom at diagnosis is hematuria (58–82%); however, approximately 8% of patients are asymptomatic (4, 5). Most patients with UrC present at advanced stages and have poor prognosis, which is partly because of the lack of specific and early symptoms (5–7). The reported 5-year overall survival (OS) rate for UrC ranges between 27% and 61% (1, 2, 4, 8, 9). No standard evidence-based diagnostic or treatment guidelines have been established for UrC owing to its rarity. Here we report a rare case of urachal adenocarcinoma (UrAC) with cervical invasion that was initially misdiagnosed as primary cervical adenocarcinoma.

## 2 Case description

In July 2020, a 63-year-old woman was admitted to the Department of Gynecology in our hospital with a bladder mass that was detected during a follow-up examination after treatment for cervical adenocarcinoma.

The patient was diagnosed with International Federation of Gynecology and Obstetrics (FIGO) stage IIIB cervical adenocarcinoma in November 2017 at a local hospital after attending because of irregular vaginal bleeding. She had a history of hypertension, and her blood pressure was well controlled. The patient was treated with concurrent chemoradiotherapy, including vaginal brachytherapy, and the last treatment was in February 2018. Then the patient underwent regular follow-ups, and no recurrence or metastasis was observed until 27 months after treatment. During a periodic examination in June 2020, magnetic resonance imaging (MRI) identified a 2.4 × 1.7 cm space-occupying lesion in the posterior wall of the bladder. The probability that the mass was a malignant tumor was considered high. Physical examination showed that the anterior vaginal wall was hard and thickened, and an irregular mass with a diameter of approximately 3 cm was discovered by palpating. The vaginal vault disappeared, and the cervix was difficult to expose. The patient was asymptomatic. Transvaginal biopsy suggested adenocarcinoma of the posterior urethral wall without tumor involvement in the cervix. A subsequent positron emission tomography-computed tomography (PET-CT) scan of the entire body revealed a high possibility of disease recurrence in the uterine cervix, posterior bladder wall, and bilateral ureteral orifices. No distant metastases were noted on the PET-CT scan. Colonoscopy and gastroscopy did not show any signs of tumors. Therefore, the primary diagnosis was considered to be the recurrence and metastasis of cervical cancer.

**Figure 1:**
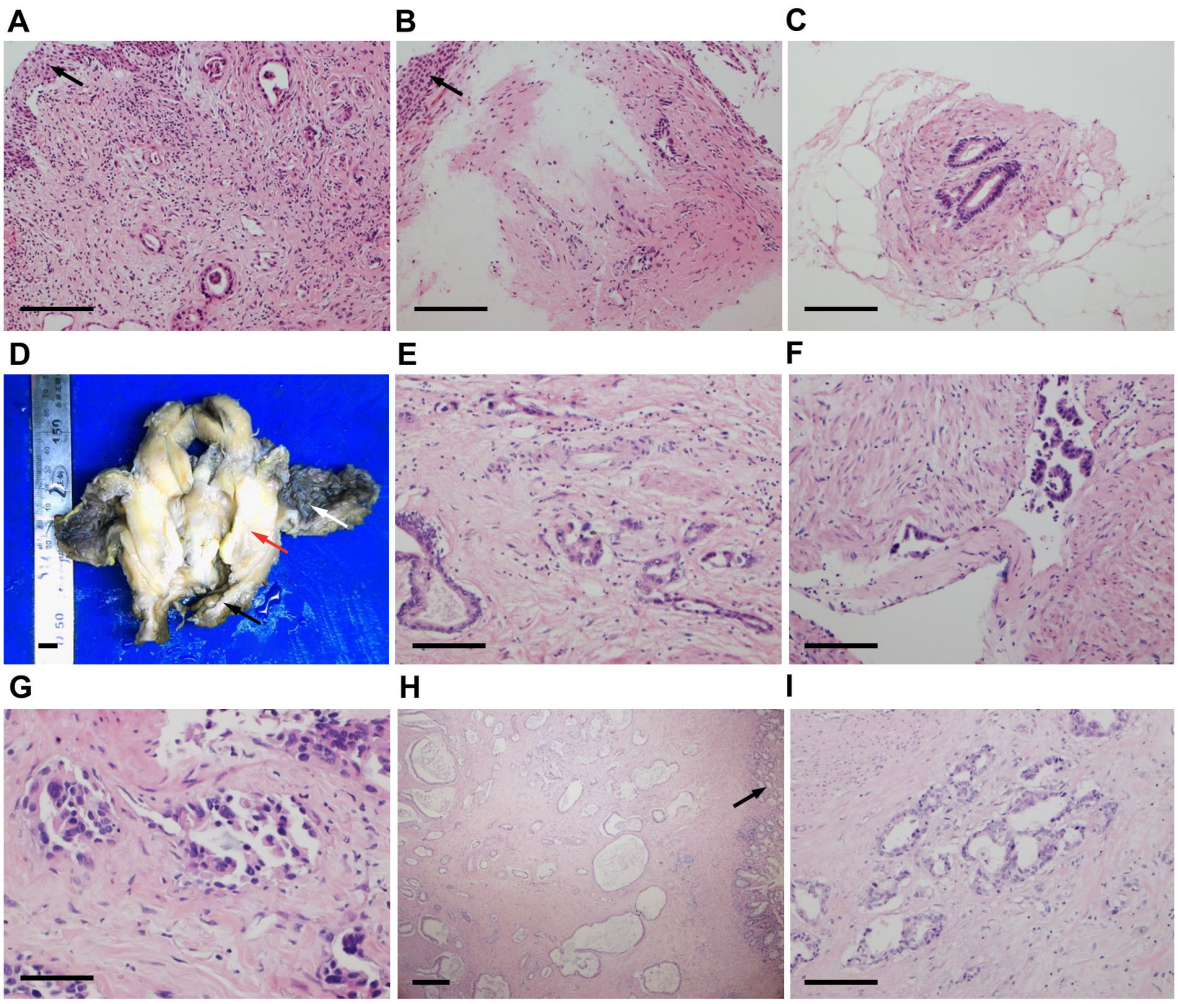
Hematoxylin and eosin (HE) staining of cervical (**A**, ×200), vaginal (**B**, ×200) and urethral (**C**, ×200) biopsy tissues. Squamous epithelium was normal (black arrows). **(D)** Surgical specimens of the bladder, uterus (red arrow), and vagina (black arrow). The bladder mucosa was normal (white arrow). HE staining of the bladder tumor (**E**, ×200) with a vascular cancer embolus (**F**, ×200). **(G)** HE staining of the puncture tissue in the thickened area of the rectum (×400). **(H, I)** HE staining of the surgical rectum specimen (**H**, ×40; I, ×200). The rectal mucosa was normal (black arrow). Similar tumor cells have been observed in cervical, vaginal, urethral, bladder, and rectal tumors, where cells are arranged in a single layer with glandular tube-like structures. Scale bars: **(A–C, E, F, I)** 400 μm. **(D)** 1 cm. **(G)** 200 μm. **(H)** 1 mm.

**Figure 2:**
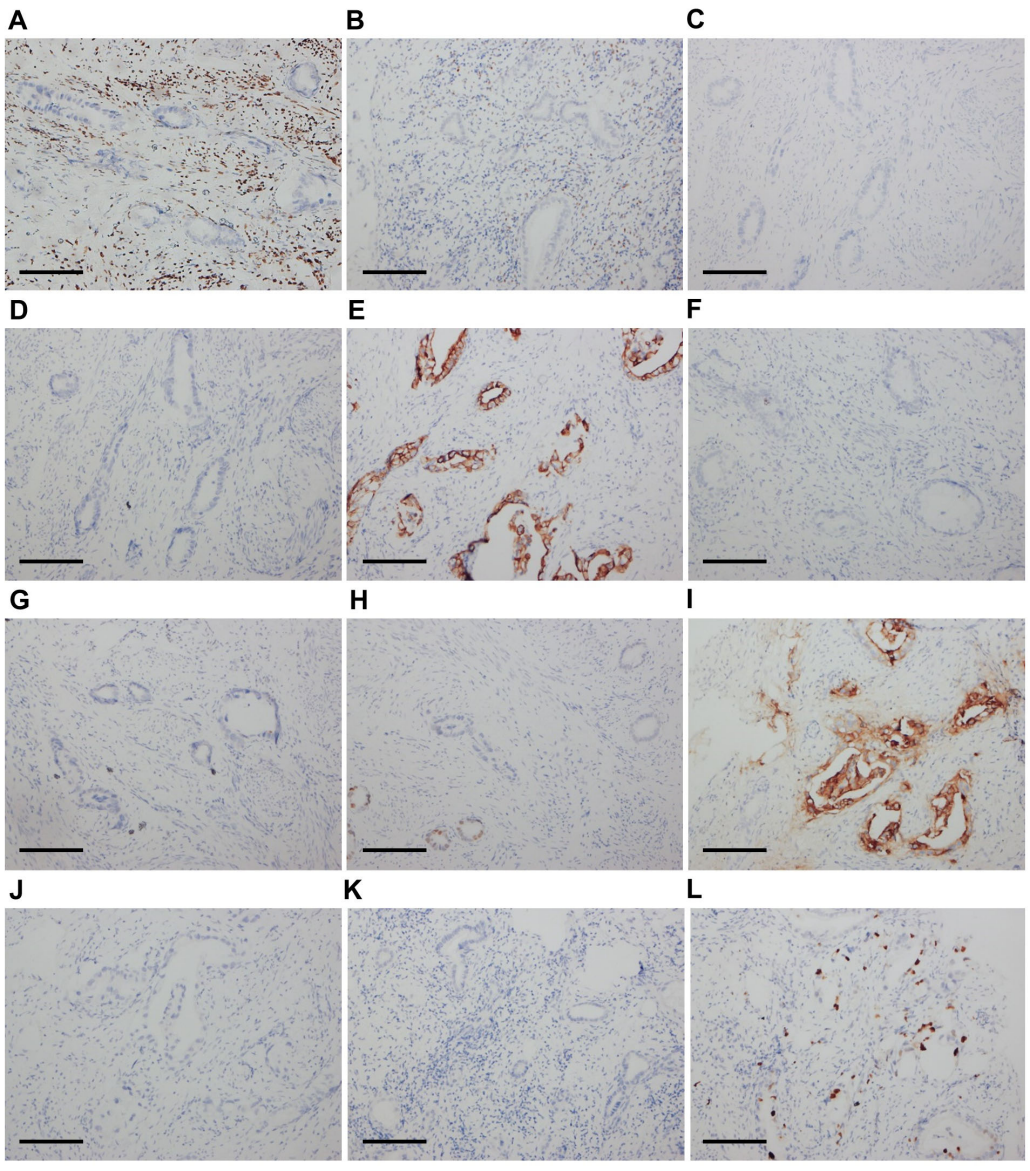
Immunohistochemical results of cervical tumor cells (×200). The cells were negative for ER **(A)**, PR **(B)**, P16 **(C)**, and CA125 **(D)**. The cells were diffusely positive for CK7 **(E)**. The cells were negative for CK20 **(F)** and SATB2 **(G)**. The cells were locally positive for CDX-2 **(H)** and diffusely positive for CEA **(I)**. The cells were negative for MUC6 **(J)** and MUC2 **(K)**. The Ki67 score was 20-60% **(L)**. Scale bars: **(A–L)** 400 μm.

Pathologists at our hospital reassessed the pathological sections that had previously been obtained by another hospital when the patient was admitted. The cervical and vaginal biopsy in 2017 indicated poorly differentiated adenocarcinoma with a vascular cancer embolus, but the squamous epithelium was normal (**Figures 1A, B**). The immunohistochemical (IHC) test revealed ER(-), PR(-), P16(-), CA125(-), CK7(+), CK20(-), SATB2(-), CDX-2(local+), CEA(+), P53(-), Napsin-A(-), CA199(+), MUC6(-), MUC2(-), and Ki67(+20–60%) (**Figures 2A–L**). Therefore, metastatic mucinous cancer was considered; however, the primary tumor remained unclear. Transvaginal biopsy performed in June 2020 confirmed adenocarcinoma of the posterior urethral wall, which was considered highly likely to be a mucinous adenocarcinoma (**Figure 1C**).

A case discussion was conducted by the gynecological oncologists to evaluate the possibility of surgical resection and prepare for anterior pelvic exenteration after communicating with the patient. During laparoscopic exploration in July 2020, a contractural lesion and tight adhesion between the posterior wall of the bladder and the anterior wall of the uterus were found, with the upper 3/4 of the vaginal wall being qualitatively hard. A laparoscopic radical hysterectomy with bilateral adnexectomy, pelvic lymphadenectomy, cystectomy, and vaginal and urethral resection were performed. The organs were removed through the vulva before vulvalplasty. Subsequently, bilateral ureterocutaneostomy was performed using two mono-J catheters as ureteral stents, and a pelvic drainage tube was inserted. The entire operation lasted 320 min, with an estimated blood loss of 300 mL, and no intraoperative complications were identified.

**Figure 3:**
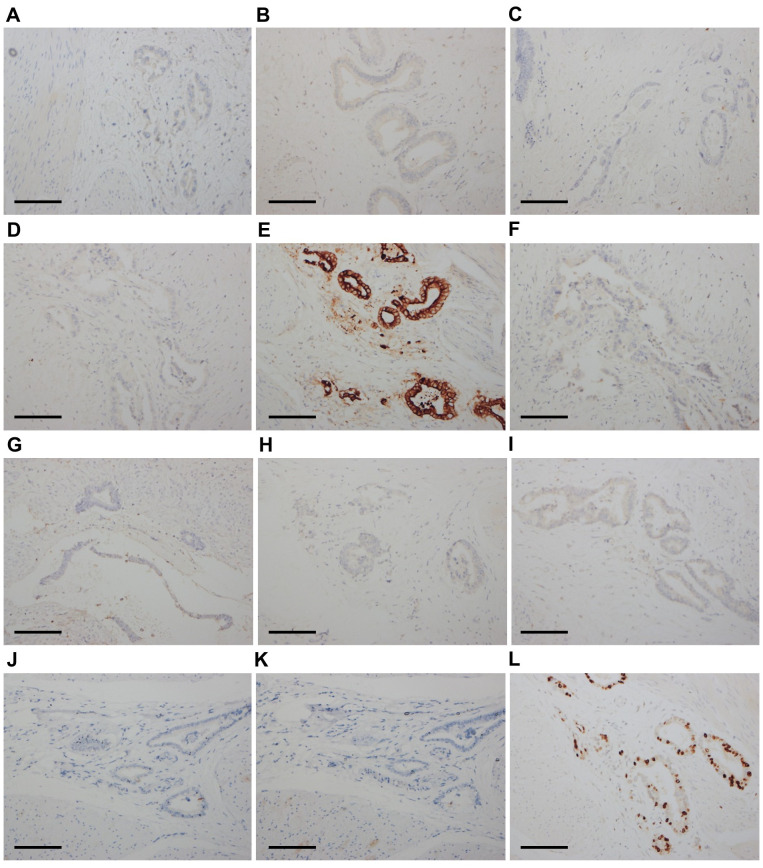
Immunohistochemical results of bladder tumor cells (×200). The cells were negative for ER **(A)**, PR **(B)**, P16 **(C)**, and CA125 **(D)**. The cells were diffusely positive for CK7 **(E)**. The cells were negative for CK20 **(F)**, SATB2 **(G)**, CDX-2 **(H)**, Pax-8 **(I)**, MUC2 **(J)**, and MUC5 **(K)**. The Ki67 score was 70% **(L)**. Scale bars: **(A–L)** 400 μm.

The final pathological analysis indicated that the lesion was a poorly differentiated mucinous metastatic adenocarcinoma that extensively infiltrated the muscular layer and outer layer of almost the entire bladder wall and the upper 3/4 of the vaginal wall interstitium. The cancer invaded some skeletal muscles outside the vaginal wall and some nerves and blood vessels in the bladder wall (**Figures 1D–F**). The bladder mucosa was normal, and no residual cancer was detected in the uterine cervix. No metastatic lymph nodes were observed, and the resected margins were negative. The IHC examination of bladder tumor cells showed CK7(+), CAM5.2(+), CA199(+), CD10(+), GATA3(local+), Ki67(+70%), and Ber-ep4(+) staining, while IHC staining was negative for ER, PR, P16, CA125, CK20, SATB2, CDX-2, Pax-8, MUC2, MUC5, Vim, P53, S-100, SMA, D2-40, Calretinin, and MC (**Figures 3A–L**). The IHC examination of vaginal tumor cells revealed CK7(+), CDX-2(+), CEA(+), CA199(+), GATA3(+), TTF-1(local+), MUC5(+), Villin(+), and Ki67(+70%), whereas IHC staining was negative for ER, PR, P53, P16, CA125, CK20, HNF1-β, Napsin-A, and MUC6. Based on the morphology, distribution characteristics of the tumors, and IHC results, the pathologists concluded that the primary cancer arose from UrAC. The clinical cancer stage was IIID UrC, according to the staging system proposed by Sheldon (10).

Regular follow-ups were conducted after surgery, and the patient underwent a tension-free hernioplasty for a pelvic floor incisional hernia in January 2021. Follow-up MRI scans were performed within 1.5 years of the anterior pelvic exenteration and showed no signs of cancer recurrence or metastasis. In March 2022, another MRI scan revealed a thickening of the middle and lower rectal walls, with an enhanced nodule on the left wall of the middle rectum. A PET-CT scan showed a high probability of metastasis to the left posterior mesentery of the middle rectum and right common iliac lymph nodes. An ultrasound-guided rectal puncture was performed, and the pathology report revealed a mucinous adenocarcinoma in the smooth muscle tissue (**Figure 1G**). IHC results revealed CK7(+), CK20(-), CDX-2(local+), SATB2(-), PAX-8(-), P16(-), HPVRNAscope(-), WT1(-), P53(wild-type), Ki67(+10%), CEA(+), PAX-2(-), ER(-), and PR(-). The immunophenotype was not aligned with HPV-associated cervical adenocarcinoma metastasis but was consistent with the metastasis of urachal mucinous adenocarcinoma.

The patient then began tumor immunotherapy with sintilimab but stopped because of severe edema in both lower extremities. From May to August 2022, the patient received four cycles of gemcitabine chemotherapy. In September 2022, after achieving a stable disease state, the patient underwent laparoscopic ultralow rectal anterior resection, ultralow anastomosis of the sigmoid colon and rectum, prophylactic transverse colostomy, and right common iliac and external iliac lymph node dissection. The postoperative pathological report indicated a highly to moderately differentiated adenocarcinoma involving the submucosa, muscular layer, and adventitia of the rectum, while the rectal mucosa was normal (**Figures 1H, I**). Based on the microscopic tumor growth pattern and the patient’s clinical history, the tumor was considered metastatic. The resected margins were negative, and no metastatic lymph nodes were observed.

**Figure 4:**
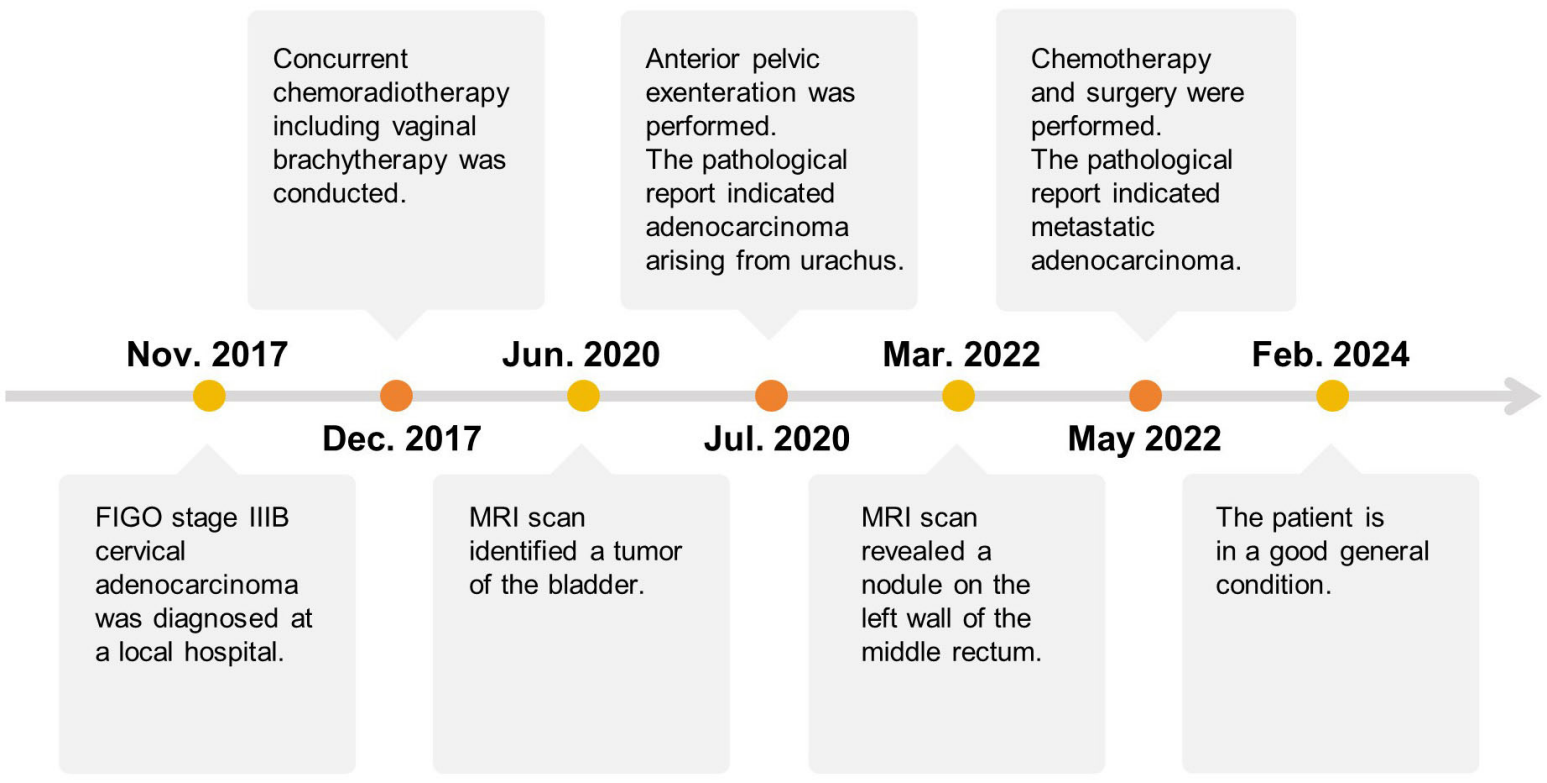
The treatment timeline of the patient.

After surgery, the patient received chemotherapy with oxaliplatin and capecitabine tablets in October 2022. However, the patient developed severe lower extremity edema and allergic reactions and refused to continue the chemotherapy. At the time of submission, the patient was continuing with regular follow-ups, and her general condition was good. The treatment timeline for the current case is shown in **Figure 4**.

## 3 Discussion

Bladder cancer is a common cancer globally, with over 600,000 new cases annually (11). Urothelial carcinoma is the most common pathological type, whereas bladder carcinomas with adenocarcinomatous features are rare and comprise 0.5–2% of cases, including primary adenocarcinoma in the bladder, UrAC, and Müllerian-type tumors (12–14). In a population-based study, including 1525 patients with primary adenocarcinoma of the bladder, UrACs accounted for approximately 10% of cases (2).

The urachus canal connects the fetal bladder and allantois. In early infancy, the urachus obliterates into a fibromuscular cord stretching between the bladder dome and umbilicus, known as the median umbilical ligament (1, 3). However, urachal remnants persist in approximately one-third of the population and carry a risk of developing various lesions (3).

In 1930, Begg published the first extensive description of UrC (15). The case reported here differs from previously reported cases of UrC because the patient had a history of cervical cancer diagnosed 2.5 years prior. When reassessing the pathological sections of the cervical and vaginal biopsies, metastatic mucinous adenocarcinoma was considered instead of primary cervical cancer. Cervical invasion or metastasis from non-gynecological cancers is relatively rare and difficult to diagnose (16). However, it is important to differentiate non-primary cervical carcinomas from primary cervical carcinomas because treatments and prognoses differ depending on the origin (17). According to the International Endocervical Adenocarcinoma Criteria and Classification (IECC), endocervical adenocarcinomas (ECAs) are classified as either HPV-associated (HPVA) or non-HPV-associated (NHPVA) (18). Mucinous ECAs comprise HPVA and NHPVA tumor subtypes (18). The former, including intestinal, signet ring cell, mucinous not otherwise specified (NOS), and invasive stratified mucin-producing carcinoma types, are usually HPV-positive (18). NHPVA mucinous ECAs refer to gastric-type adenocarcinomas (18), which usually stain positive for CK7, CEA, CA125, MUC6, CA199, and HNF1-β (19). The pathological results of the tumor specimens in this study showed that the mucinous adenocarcinoma was P16-negative, and *in-situ* hybridization with the RNAscope probe showed that it was HPV-negative. Thus, a differential diagnosis to exclude NHPVA cervical adenocarcinoma, particularly gastric-type carcinoma, was considered in this case. Consequently, the described case was reviewed and discussed by oncologists and pathologists. Similar tumor cells have been observed in cervical, vaginal, urethral, rectal, and bladder tumors arranged in a single layer with glandular tube-like structures. The nuclei were mildly to moderately atypical, with occasional mitotic figures. The squamous epithelium was normal in the cervical and vaginal biopsy tissues collected in 2017, and the mucosae of the bladder and rectum sampled in the surgeries were also normal. The IHC examination of tumor cells revealed CK7(+), CEA(+), and CA199(+), whereas IHC staining was negative for ER, PR, P53, P16, CA125, CK20, HNF1-β, and MUC6. The patient was initially diagnosed with stage IIIB cervical cancer six years prior to this report; however, the tumor was always confined to the pelvis, and the patient’s general condition was good during treatment. These findings are consistent with the pathomorphological, immunohistochemical, and clinical characteristics of UrAC rather than those of primary cervical adenocarcinoma. After discussion, the patient was diagnosed with primary advanced UrAC. We determined that the patient had been misdiagnosed as having primary cervical adenocarcinoma in 2017, and the correct diagnosis would have been urachal mucinous adenocarcinoma with cervical invasion.

UrC is a rare malignancy, and there is still no consensus on diagnostic criteria and standard treatment (1). Several diagnostic criteria for UrCs were proposed in 1954 (20, 21). In 2016, Paner et al. modified the UrAC criteria to include the following characteristics: (a) tumor located in the dome/anterior wall of the bladder; (b) epicenter of carcinoma in the bladder wall; (c) absence of a urothelial bladder tumor; (d) absence of widespread atypical intestinal metaplasia, cystitis/glandularis beyond the dome/anterior wall; and (e) absence of primary adenocarcinoma of a different origin that has spread secondarily to the bladder (3). However, some researchers consider these criteria overly restrictive. For example, Arlene et al. emphasized that urethral cancers occur anywhere along the urachal ligament. Although it occurs in most cases, bladder involvement is not medatory at the time of diagnosis (22). Further, urachal remnants are observed not only at the bladder dome but also on the anterior or posterior wall along the midline in surgical series (23). In our case, the cancer invaded almost the entire bladder wall at admission to our hospital, meeting the diagnostic criteria for UrC on postoperative pathology.

Diagnosing UrC before surgery is challenging. Abdominal ultrasound can detect the masses in the bladder wall. Computerized tomography (CT) scans and MRIs can evaluate local invasions, lymph nodes, or distant metastases (4, 24). Cystoscopy aids in tumor localization and biopsies of the mass (4, 24). Szarvas et al. (24) found that urinary cytology presented as positive in only 29% of cases. Siefker-Radtke et al. (25) explored the potential role of serum markers in a cohort of 42 UrC and reported an increase in CEA (59%), CA19-9 (60%), and CA125 (44%). Meeks et al. (26) assessed the preoperatively available methods for the diagnosis of UrC, such as biopsy by transurethral resection, imaging, cytology, and exploration under anesthesia. Biopsy had the highest specificity (1) and positive predictive value (1), with a sensitivity and negative predictive value of 0.93 and 0.5, respectively. Combining imaging and biopsy did not offer a high negative predictive value (0.60). Therefore, reliable preoperative UrC diagnosis methods are currently lacking (26).

In our case, when the cervical mass was initially detected, an MRI did not reveal any suspicious tumor lesions beyond those in the cervix and vagina. However, due to limited MRI sensitivity and biopsy specimen content, ruling out urachal mucinous adenocarcinoma with cervical invasion was difficult. We feel that oncologists and pathologists should consider non-primary cervical carcinoma, especially when cervical biopsy tissue is limited. A thorough workup for differential diagnosis is necessary for patients with uncommon presentations or pathological findings for cervical adenocarcinoma. In addition, a comprehensive analysis is crucial, as imaging and histopathological findings may not be definitive.

Adenocarcinoma is the most common type of UrC and shares similarities with adenocarcinomas of other origins, particularly colorectal adenocarcinomas (4, 27). Usually, IHC is crucial for differential diagnosis; however, in some cases, it cannot provide an accurate diagnosis because of overlapping tumor features. In UrAC, CK20 is positive in approximately 97% of cases, while CK7 is positive in 51% (27). CDX2 and MUC5AC are often detected at high rates in UrACs (90% and 92%, respectively), and nuclear staining with β-catenin is found to be positive in 14% of cases (27). According to Bayrak et al., the CK7-/CK20+ immunophenotype is a specific and sensitive marker of colorectal origin. The CK7+/CK20- phenotype was expressed in only 1.7% (2 of 118) of colorectal adenocarcinomas (28). Diffused CK7 and β-catenin can help differentiate the enteric subtype of UrAC from colonic adenocarcinoma, with the former being nuclear β-catenin(-) and CK7 (+/-), while the latter being diffuse nuclear β-catenin(+) and CK7(-) (3, 27, 29). In our case, the CK7+/CK20- phenotype and intact normal colorectal mucosa did not support primary colorectal adenocarcinoma.

The occurrence and development of bladder urothelial carcinoma are reported to be associated with DNA-level molecular alterations (30, 31). Additionally, the most frequently mutated genes in ordinary bladder cancer are KMT2C, ATM, FAT1, CREBBP, ERBB2, SPTAN1, and KMT2A (30, 31). However, several molecular analyses in previous studies have shown that the mutation pattern of UrC is more similar to colorectal than to urothelial carcinoma (4, 32, 33). Mutational hotspots of selected genes were tested in 22 UrC samples, and KRAS mutations in 6 of the 22 (27%) UrC cases were found, followed by BRAF (18%) and NRAS (5%). No mutations in the PIK3CA and EGFR genes were observed (32). In a research study on 17 patients with UrC by Lee et al., they found that six genes in functionally important domains had recurrent mutations: COL5A1, APC, LRP1B, SMAD4, KIF26B, and TP53. In addition, the fibroblast growth factor receptor (FGFR) gene family was amplified in six patients, while the epidermal growth factor receptor (EGFR) family was amplified in four (33). These findings also suggest that potential treatments for urachal cancer should target these gene families; for example, anti-EGFR agents and FGFR inhibitors (33).

Surgery is the recommended therapy for localized cases of UrC. Excision of the urachus, the umbilicus, partial/radical cystectomy, and bilateral pelvic lymphadenectomy are standard procedures and thus routinely performed (34). Research indicates that survival outcomes do not significantly differ between patients treated with either partial cystectomy or radical cystectomy procedures (35, 36). However, UrAC often recurs (20–54%) post-surgery, and the median time to recurrence after resection of the primary tumor is reported to be approximately 29–32.7 months (4, 25, 37). Chemotherapy, with cisplatin and 5-FU, is the most commonly used regimen for treating metastatic disease (4). A meta-analysis showed that cisplatin-5-FU combination therapy might be the most effective treatment as it shows a high (43%) response rate and a low (14%) progression rate (24). The similarity of urachal cancer to colorectal adenocarcinoma has led some researchers to propose several schemes that are reasonable for small sample sizes, such as mFOLFOX6 (a modified combination of 5-fluorouracil, leucovorin, and oxaliplatin), cetuximab, and irinotecan (38–40). For example, Yanagihara et al. stated that mFOLFOX6 appears to be effective for the treatment of metastatic urachal cancer, with one of the five patients (20%) achieving a clinically complete response and another one achieving a partial response (40). Collazo-Lorduy et al. (38) reported a patient with metastatic urachal cancer who was EGFR amplified and had wild-type KRAS. The patient was treated with cetuximab, an anti-EGFR monoclonal antibody, and achieved a partial response for more than eight months (38). However, multicenter collaborations are required to validate these drug treatments for this rare malignancy.

Pelvic exenteration is an ultra-radical surgery that involves complete en bloc resection of malignant lesions and pelvic viscera, divided into anterior, posterior, and total pelvic exenterations (41). It is the only curative option for many locally advanced and recurrent pelvic malignancies after patients have previously undergone chemotherapy, radiotherapy, or surgery (42). In our case, anterior pelvic exenteration was performed before a clear diagnosis. The procedure seems to be effective against the local pelvic recurrence of UrC. However, as only one case was evaluated, further investigations, including prospective data, are needed to validate the efficacy of the procedure.

## 4 Conclusions

UrC is a rare malignancy that is diagnostically and therapeutically challenging for oncologists, and accurate histopathological examinations and comprehensive analyses are essential to avoid misdiagnoses. In this report, anterior pelvic exenteration was identified as a potentially effective treatment for a patient with a local pelvic recurrence of UrC. However, further study of existing treatment methods is required to establish a standard treatment strategy. To the best of our knowledge, this is the first report of a patient with UrAC misdiagnosed as primary cervical cancer, and we hope to provide further reference material to improve the diagnosis and treatment of this disease.

## Data Availability

The original contributions presented in the study are included in the article/supplementary material. Further inquiries can be directed to the corresponding authors.
